# Correction: Effect of caffeine on neuromuscular function following eccentric-based exercise

**DOI:** 10.1371/journal.pone.0230470

**Published:** 2020-03-11

**Authors:** Ana C. Santos-Mariano, Fabiano Tomazini, Leandro C. Felippe, Daniel Boari, Romulo Bertuzzi, Fernando R. De-Oliveira, Adriano E. Lima-Silva

The image for Fig 4, “Countermovement jump test performed before and after a half-squat exercise and at 24, 48 and 72 h after placebo or caffeine ingestion,” is incorrect. Please see the correct Fig 4 here.

**Fig 4:**
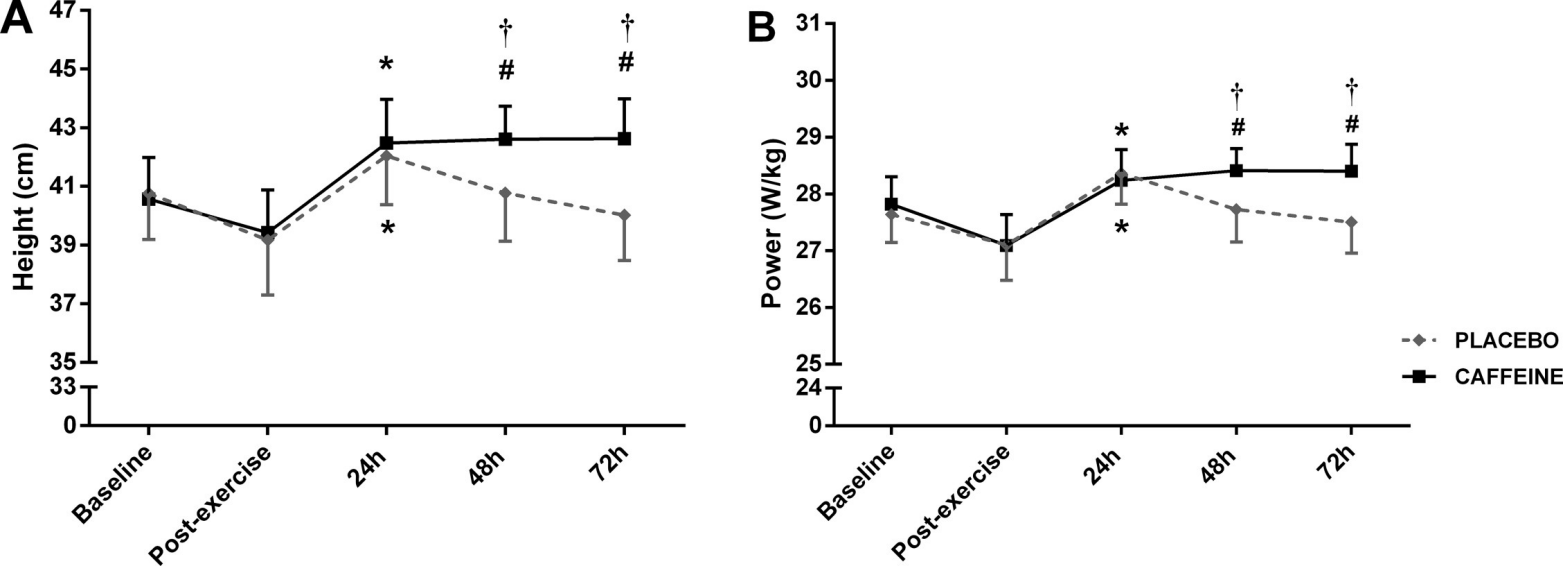
Countermovement jump test performed before and after a half-squat exercise and at 24, 48 and 72 h after placebo or caffeine ingestion. Values are expressed as mean ± SEM. A: Height; B: Power (relative to body mass). *Significantly higher than pre- and post half-squat exercise in both conditions; ^#^Significantly higher than pre- and post half-squat exercise only in caffeine condition; ^†^Significantly higher than placebo at the same time point.
