# Supplementary material for: Effect of caffeine on neuromuscular function following eccentric-based exercise
Source: PLoS One. 2019 Nov 7;14(11):e0224794. doi: 10.1371/journal.pone.0224794 (PMC6837311; doi:10.1371/journal.pone.0224794)
Supplement: Attachment — Submitted filename: REVISION.docx [file pone.0224794.s001.docx]

Manuscript Number: PONE-D-19-16976

**Effect of Caffeine on neuromuscular function following eccentric-based exercise**

General Comments

Authors carried out an interesting research regarding to the effect of caffeine on recovery of neuromuscular function, power and sprint performance following an eccentric-based exercises using a "*Tirante Muscular®* " in half squat exercise. Authors did a great job controlling many variables providing a broad vision when studying central and peripheral fatigue, DOMS, perceived recovery, muscle damage, CMJ and Sprint. However, the manuscript is well written and very good organized. Should be highlight why the *Tirante Muscular®* has been used, since it is not clear the reason that you chose this tool and not a Smith Machine or free weight. On the other hand, in the paragraph of experimental design, authors describe the sprint pre-test as “one sprint of 30 m, one of 50 m and one of 100m”. In the post- test, has not been carried out the same procedure, conversely have been performed: “1) 3 sets of 30m sprints, with a 5min rest between sets; 2) 3 sets of 50 m sprints, with a 7 min rest between sets and; 3) 3 sets of 100 m sprints, with a 10 min rest between sets”. These two protocols are different and the results cannot be compared due to the volume and the fatigue in each test. It is a limitation of this study that should be shown in the manuscript. It is possible to think that the performance in the sprint has not improved due to the fatigue that the post-test supposes. Perhaps a single series of 30m would have been enough to compare the performance in the sprint effectively. By this is important that authors explain it. Finally, only some changes I can propose to improve this manuscript. Thus, I considered that the paper could be accepted if the authors could correct mistakes pointed out. My specific comments are presented below.

Specific Comments

Page 2, line 42: Before “and”, “,” should not be used. On the other hand, between “72” and “h” the “-” it is not necessary and the authors due write in the same form along the manuscript because in some case don’t use hyphen.

Page 2, line 45: The use of concept baseline is not appropriated to refer to Sprint performance. This concept is well used to muscle damage, DOMS or RT, but sprint need a warm up and activation process to do the best performance. By this, change “Baseline” by “Pre-test” could be more appropriated.

Page 2, line 48,52: Remove the hyphen (24 h) (48 h) (72 h).

Page 3, line 73: It would be good to write a brief description of how DOMS is measured in the literature.

Page 3, line 73,77,78: Remove the hyphen (24 h) (48 h) (72 h).

Page 4, line 101: It is necessary write the name of Creatine Kinase before (CK) since is the first time that appear this concept during de manuscript.

Page 5, line 111-112: After “years old” should be used colon not comma and it is necessary write “height:” before 180.7…. Also, it is interesting to report the Mean and SD of 1RM load or the load used corresponding with 70%1RM.

Page 5, line 123: The sentence starts very similar like the sentence before. Change “study” by “research” could be a good strategy of avoid be repetitive.

Page 5, line 126: Explain if the 1RM test was carried out with *Tirante Muscular®* .

Page 6, line 133-134: Unify the way to refer days (with numbers “2” or letters “two”).

Page 6, line 134: Change “baseline” by “Pre”. Also, before “and”, “,” should not be used, remove it.

Page 6, line 134-135: How much time was used to recover between the sprints?

Page 6, line 134-137: Was carried out some warm up before Sprints and CMJ? Could you explain it?

Page 6, line 139: Explain if the half-squat exercise was performed with *Tirante Muscular®.*

Page 6, line 157: Remove the hyphen (48 h).

Page7, line 173,175: Remove the hyphen (2 min).

Page 8, line 182,183: Remove the hyphens (2 min) (3 s).

Page 8, line 193: Remove the hyphen (0.5 min).

Page 8, line 202: Remove the hyphen (30 s).

Page 9, line 209,211,212,216: Remove the hyphens.

Page 10, line 240: Could you explain the stretching exercises that was carried out? It is known that passive stretching can impair the performance.

Page 11, line 251: Explain if a nurse or another qualified professional took the serum extractions.

Page 11, line 252: You have used “Creatine Kinase” before, the abbreviation is enough in this case.

Page 11, line 258, 260: Unify the way to refer the numbers of the scale (with numbers “0” or letters “zero” and “six”). Change words by numbers.

Page 12, line 284,285: Before “and”, “,” should not be used, remove it.

Page 12, line 289: If you have used “a half-squat exercise” during all manuscript in this case is more appropriate than “a half-squat resistance training”. Change it.

Page 13, line 299: Before “and”, “,” should not be used, remove it.

Page 13, line 312: Add “exercise” after a half squat.

Page 14, line 333: You can use “CMJ” instead of “countermovement jump”

Page 14, line 334: If you have used “a half-squat exercise” during all manuscript in this case is more appropriate than “a half-squat resistance training”. Change it.

Page 15, line 346: If you have used “a half-squat exercise” during all manuscript in this case is more appropriate than “a half-squat resistance training”. Change it.

Page 16, line 368: Add “s” after “athletes’ ”

Page 16, line 378: Remove the hyphens (48 h) and (72 h)

Page 17, line 392: It would be interesting to complete the paragraph with a section of limitations, perhaps report about the pre and post sprints test or regarding to only use one caffeine intake protocol.

Figures 3 and 4: Unify the levels of values in the Y axis of all figures. MVC has nine levels, Q_twpost_ has seven levels or Q_tw100_ has eight levels.
