# Supplementary material for: Effect of caffeine on neuromuscular function following eccentric-based exercise
Source: PLoS One. 2019 Nov 7;14(11):e0224794. doi: 10.1371/journal.pone.0224794 (PMC6837311; doi:10.1371/journal.pone.0224794)
Supplement: Attachment — Submitted filename: PONE REVIEW.docx [file pone.0224794.s002.docx]

Review to: PONE-D-19-16976; Effect of caffeine on neuromuscular function following eccentric-based exercise.

Major comments.

This experiment was designed to assess the neuromuscular effects of caffeine intake during the recovery phase of an eccentric-based exercise protocol that induces muscle damage. Overall, the experiment seems well designed, it contains several measurements to fulfill the objectives of the investigation and it is conveniently described in a well-written manuscript. However, the experiment has a serious flaw: it is designed to assess caffeine’s effects in the recovery phase of a muscle-damaging exercise when most, if not all, performance variables returned to basal -non-fatigued- values within 24 hours after the end of exercise. This fact is likely due to the use of a “light” eccentric exercise protocol that produced very low levels of muscle damage – a pilot study would have been helpful to detect the inefficacy of this protocol to produce moderate muscle damage”.

Thus, although I found merit in the experiment and I congratulate authors for the hard work developed for this investigation, I would suggest that authors reconsider the use of “recovery” through the manuscript because this is not what they were investigating -the recovery lasted < 24 h and they did not perform any measurement in this time. For example, the conclusion of the abstract should indicate that:

“Caffeine improves muscle power 48 and 72 hours after an eccentric-based exercise…”.

A limitation paragraph including this aspect is highly recommended.

Other comments.

Introduce information about the use of caffeine in athletics. Is this common?

Indicate why you selected this protocol of eccentric exercise. And discuss the lack of a relevant increase in CK (for instance, you could compare these values with the ones found after a marathon).

Indicate the habituation to caffeine of these individuals.

The CK concentrations before exercise seems quite high (especially one participant with > 500 U/L). Please, discuss this in the light of previous investigations. Did they have previous muscle damage due to training?

Why there is no data on jump height?

Please, indicate the calculation of the sample size or at least, the statistical power with this sample of 11 individuals.

A paragraph of practical applications for jumpers and sprinter might be suitable. How they should use caffeine during their training. Before all sessions? 48 h after a high-intensity session?

Line 397. “sprinters” is twice.
